# Integrative bibliometric and transcriptomic analyses identify selenium-associated molecular signatures in the aging brain

**DOI:** 10.3389/fnagi.2026.1791352

**Published:** 2026-05-04

**Authors:** Shan Liu, Bo Yan, Han Gao, Lin Zhang, Zihan Zhang, Yaru Liu, Fanglian Chen, Ping Lei

**Affiliations:** 1 Department of Geriatrics, Tianjin Medical University General Hospital, Tianjin, China; 2 Key Laboratory of Post-Trauma Neuro-Repair and Regeneration in Central Nervous System, Ministry of Education, Tianjin Key Laboratory of Injuries, Variations and Regeneration of Nervous System, Tianjin Neurological Institute, Tianjin Medical University General Hospital, Tianjin, China; 3 Department of Gastroenterology, The Central Hospital of Enshi Tujia and Miao Autonomous Prefecture, Enshi, China; 4 School of Medicine, Nankai University, Tianjin, China

**Keywords:** bibliometrics, brain aging, machine learning, selenoproteins, SEPHS2, SP1

## Abstract

**Background and purpose:**

The aging brain is particularly sensitive to alterations in selenium status. Selenium deficiency has been associated with impaired neural function, cognitive decline, and increased vulnerability to neurodegeneration. However, the molecular mechanisms that link selenium biology to brain aging remain poorly understood.

**Methods:**

We conducted a bibliometric analysis of 1,826 publications and identified brain-aging DEGs from public datasets. After intersecting these with selenium-related gene sets, we used machine-learning feature selection and SHAP/nomogram evaluation to prioritize core genes, validated findings in an independent cohort, performed immune-infiltration and gene-drug enrichment analyses, and confirmed age-related transcriptional and protein changes in mouse brain tissue.

**Results:**

Bibliometric analysis showed a steady increase in publications on selenium and aging over the past two decades, with major research hotspots focusing on oxidative stress, selenoproteins, and cognitive function, while the selenium-cognition relationship remains relatively underexplored. Intersection analysis identified seven potential targets linking selenium to brain aging, from which machine-learning feature selection prioritized three core genes (SP1, SEPHS2, and MSRB1) that were significantly differentially expressed in aged samples. SHAP and nomogram analyses indicated that SP1 and SEPHS2 were the main contributors to model discrimination. Animal experiments further confirmed increased SP1 and decreased SEPHS2 expression at both mRNA and protein levels in aged mouse brains, consistent with the bioinformatic findings.

**Conclusion:**

This study identifies SP1 and SEPHS2 as key genes linking selenium to brain aging, providing new insights into the role of selenium in brain aging and suggesting that these genes may represent potential biomarkers or therapeutic targets for brain aging and aging-related brain disorders.

## Graphical Abstract

**Figure:**
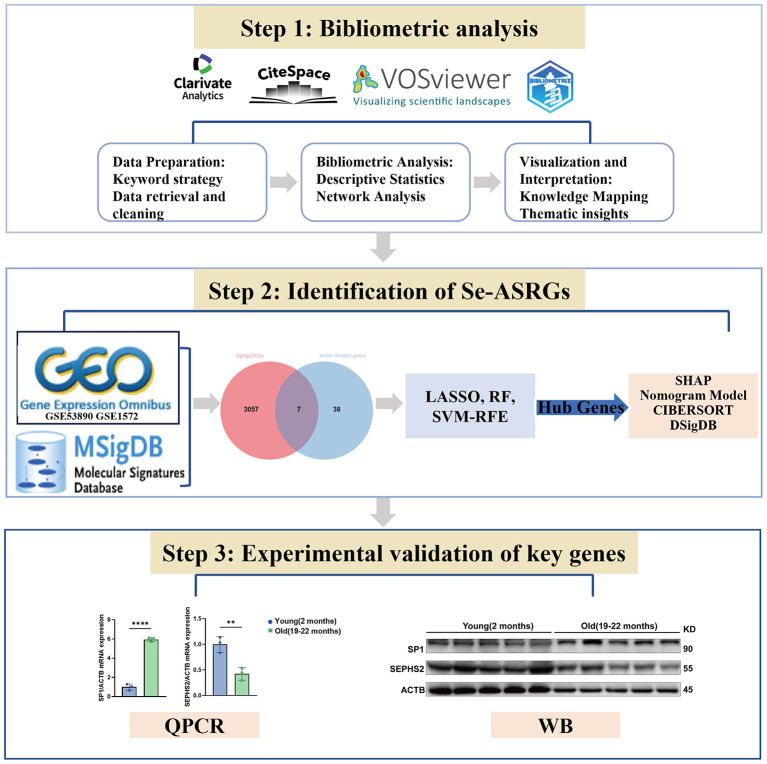
This study integrates bibliometric analysis, transcriptomic profiling, machine learning-based feature selection, and experimental validation to identify key selenium-associated molecular features involved in brain aging, highlighting SP1 and SEPHS2 as key regulatory genes.

## 1 Introduction

Population aging, driven by increasing life expectancy, poses a significant public health challenge (Beard et al., 2016). Aging is strongly linked to an increased risk of neurodegenerative, cardiovascular, and malignant diseases (Guo et al., 2022). At the cellular and molecular levels, this age-related functional decline is characterized by the progressive deterioration of physiological function and tissue integrity, accompanied by hallmark processes including genomic instability, mitochondrial dysfunction, impaired autophagy, stem-cell exhaustion, chronic inflammation, loss of proteostasis, dysregulated nutrient sensing, and altered intercellular communication (Kroemer et al., 2025). Notably, trace elements play crucial roles in maintaining cellular and molecular homeostasis during aging by modulating redox balance, serving as enzyme cofactors, and regulating aging-related signaling pathways (Farag et al., 2021). Longitudinal data from the EPIC-Potsdam cohort have documented coordinated age-related changes in circulating trace elements, notably declines in manganese, zinc, and selenium (Se), alongside increases in iron, copper, and iodine (Baudry et al., 2020). Among these, Se is an essential micronutrient with pivotal roles in maintaining multisystem homeostasis and regulating aging-related mechanisms (Zhang et al., 2023). Although interest in selenium-dependent molecular pathways has grown substantially, a comprehensive synthesis delineating research trends, thematic connections, and the field's knowledge architecture remains lacking.

Dysregulation of selenium homeostasis, reflected by reduced serum selenium levels and dysregulated selenoprotein expression, has been associated with reduced lifespan (Akbaraly et al., 2005), cognitive decline, and an increased risk of neurodegenerative diseases (Hambright et al., 2017), which may be mediated by impaired antioxidant defense (Lee et al., 2025), mitochondrial dysfunction (Wesolowski et al., 2022), and altered neuroinflammatory responses (Huang et al., 2012). Notably, while the liver, kidney, and skeletal muscle account for the majority of systemic selenium reserves (Zhang et al., 2023), brain selenium levels are selectively maintained under selenium-deficient conditions, underscoring the high physiological importance of cerebral selenium homeostasis. This selective maintenance is largely mediated by the transport of liver-derived selenoprotein P across the blood-brain barrier via the ApoER2/LRP8 receptor system (Kryukov et al., 2003; Solovyev et al., 2021). Moreover, SELENOP-independent local regulatory mechanisms have also been reported to contribute to the maintenance of brain selenium homeostasis (Schomburg et al., 2003; Schweizer et al., 2005). Such preferential preservation of cerebral selenium homeostasis sustains key selenoproteins that maintain neuronal integrity (Hambright et al., 2017; Solovyev, 2015). However, despite the growing body of research on selenium-related proteins in brain aging, the evidence remains fragmented. Mechanistic investigations remain largely limited to *in vitro* and animal studies (Raschke et al., 2023), making direct extrapolation to human biology challenging. Moreover, observational studies have reported inconsistent findings regarding dose-response relationships (Hu et al., 2019), while randomized controlled trials have also yielded heterogeneous results (Rita Cardoso et al., 2016). More importantly, systematic molecular evidence derived from transcriptome analyses of the aging human brain is still lacking. This gap has hindered in-depth exploration of the molecular basis underlying selenium homeostasis dysregulation during brain aging and has limited a comprehensive understanding of selenium's role in this process.

In this study, we established an integrative framework combining bibliometric and bioinformatic analyses. Bibliometric approaches were applied to characterize the global research landscape and thematic evolution of selenium- and aging-related studies, identifying brain aging as a key underexplored area. Subsequently, bioinformatic analyses were used to identify selenium-related genes altered in the aging brain and to evaluate their predictive, immunological, and translational relevance. Together, we identify SP1, SEPHS2, and MSRB1 as putative selenium-associated aging hubs in the aging brain; these genes may represent candidate targets for modulating selenium biology and could potentially serve as biomarkers of cerebral selenium homeostasis or molecular signatures of selenium-related brain aging.

## 2 Methods

### 2.1 Data source

On December 25, 2025, a systematic literature search was conducted in the Science Citation Index Expanded (SCI-E) database of the Web of Science Core Collection (WoSCC) (https://www.webofscience.com/). A topic search strategy was applied to titles (TI), abstracts (AB), and author keywords (AK). Search terms related to Se and aging were comprehensively combined using standardized medical terminology, Medical Subject Headings, and relevant synonymous expressions. The full search strategy is provided in Supplementary Table S1. The search was restricted to publications between 2005 and 2025. Only English-language original research articles were included, while reviews, letters, conference proceedings, and other non-research document types were excluded to ensure methodological rigor and consistency. After removing duplicate records, 1,826 publications met the inclusion criteria and were included in the subsequent bibliometric analysis.

### 2.2 Bibliometric analysis

After identifying eligible publications based on the inclusion criteria, bibliometric analyses were performed using CiteSpace (version 6.4.R2, https://citespace.podia.com/), VOSviewer (version 1.6.20, https://www.vosviewer.com/), and R software (version 4.2.3, https://www.r-project.org/) with the bibliometrix package (version 4.2.1, https://www.bibliometrix.org/).

VOSviewer was used to perform co-occurrence and co-citation analyses of authors, institutions, countries, and journals to characterize collaboration patterns and the distribution of publication output. In particular, a dual-threshold strategy was employed for institution-level analyses: institutions were required to meet the publication threshold and to have a total citation count of ≥300 to be included, thereby improving the robustness and interpretability of the institution network. For journal-level analyses, two complementary approaches were applied to capture different aspects of journal activity: (1) to define active outlets publishing on selenium and aging, a minimum publication threshold of ≥5 publications per journal was used; (2) to identify the field's most influential journals, a journal co-citation network was constructed using a minimum citation threshold of ≥200. The citation cutoff for the co-citation analysis was selected to reduce noise from low-impact nodes and to focus the network on highly cited, field-relevant core journals, consistent with previously published methodological recommendations (van Eck and Waltman, 2010; Donthu et al., 2021). Moreover, except for the parameters emphasized above, all other parameters were set to VOSviewer's default values in the analyses, and detailed information on all parameters is available in the VOSviewer user manual. Keyword co-occurrence networks were constructed to depict the thematic structures, core research topics, and their temporal evolution in the included studies, with a minimum keyword occurrence threshold of 20. Before network construction, keywords from all eligible publications were extracted and standardized: synonymous terms, abbreviations, and spelling variants were unified, and duplicate entries were removed to ensure data consistency. The cleaned keyword dataset was used to construct the subsequent network. Moreover, to highlight selenium-related research in brain aging, keyword subnetworks centered on “brain” and “aging” were extracted from the keyword co-occurrence matrix to examine their functional roles within the global research network.

CiteSpace was applied to build co-citation networks and perform timeline clustering analyses, with years as time slices (1 year per slice) and the g-index (k = 25) for node selection. The built-in log-likelihood ratio algorithm was adopted to generate thematic clusters, with all other parameters set to their default values. Timeline views and dual-map overlays were further used to visualize the evolutionary trends in research themes and the temporal progression of citation relationships over the study period. Conceptual structure and thematic evolution maps were generated using Bibliometrix. Multiple correspondence analysis (MCA) was used to visualize the distribution of keywords in the semantic space, and thematic strategy maps based on centrality and density metrics were constructed to evaluate the research relevance and developmental stage of different thematic clusters.

### 2.3 Identification of aging-related DEGs and selenium-related genes

The gene expression data (human frontal cortex) from the GSE53890 (Lu et al., 2014) dataset was obtained to serve as the training set for this study, while the GSE1572 (Lu et al., 2004) dataset was used as the validation set. Differential expression analysis was performed between the young group (< 60 years old) and the old group (>60 years old). Based on predefined statistical thresholds (adjusted *P*-value < 0.05 and |log_2_ fold change| ≥ 0.2), a set of aging-related differentially expressed genes (DEGs) was identified. KEGG pathway and GO enrichment analyses were conducted via the R package clusterProfiler, with a significance threshold set at *P* < 0.05. Selenium metabolism—and selenoprotein-related gene sets were obtained from MSigDB (MSigDB, v2025.1.Hs, https://www.gsea-msigdb.org/gsea/msigdb), and a protein-protein interaction (PPI) network was constructed using the STRING database (version 12.0, https://string-db.org/) to characterize Se-ASRGs.

### 2.4 Machine learning-based feature selection of Se-ASRGs

Least absolute shrinkage and selection operator (LASSO) regression was conducted using the glmnet package to reduce feature dimensionality, with the optimal regularization parameter (λ) determined using 5-fold cross-validation. Genes associated with non-zero coefficients were retained for subsequent analysis. In parallel, a random forest (RF) model consisting of 500 decision trees was established to assess the relative importance of candidate genes. Additionally, support vector machine-recursive feature elimination (SVM-RFE) (Nedaie and Najafi, 2018) was performed using the “e1071 ” package to screen genes with strong discriminative potential further. Genes consistently identified by all three machine learning approaches were defined as hub selenium-associated aging-related genes (hub Se-ASRGs).

### 2.5 SHAP analysis

Hub selenium-associated aging-related genes (Hub Se-ASRGs) were used to construct multiple machine learning models for the classification of aging status, including logistic regression (LR), random forest (RF), decision tree (DT), k-nearest neighbors (KNN), glmBoost, partial least squares (PLS), extreme gradient boosting (XGBoost), and neural network (NN) models. Model performance was evaluated based on the area under the receiver operating characteristic curve (AUC), and the model with the highest AUC was selected for Shapley Additive exPlanations analysis. SHAP values were calculated to quantify the contribution of each hub Se-ASRG to model predictions and to rank their relative importance.

### 2.6 Construction and evaluation of an aging prediction model

A Binary LR model was employed to develop an aging prediction model. Specifically, this LR model was utilized to determine the matching coefficients of hub Genes and calculate the risk score for each sample. To improve its clinical applicability, the R package “rms” was adopted to construct a predictive nomogram, which incorporated the pivotal SHAP-identified Hub genes. Calibration curves, decision curve analysis (DCA), and AUC value evaluation were applied to verify the accuracy and reliability of the established nomogram model. Furthermore, the robustness and generalizability of the model were strengthened through validation on the external dataset GSE1572.

### 2.7 Immune cell infiltration analysis

The CIBERSORT algorithm was applied for the deconvolution of bulk transcriptomic data to estimate immune cell composition based on gene expression profiles. Spearman's rank correlation analysis was performed to examine the relationships between hub selenium-associated aging-related genes and immune cell infiltration patterns. The correlations between gene expression levels and immune cell fractions were visualized using the R package linkET.

### 2.8 Drug enrichment analysis

Drug enrichment analysis was performed to evaluate the potential pharmacological relevance of Se-ASRGs by querying the DSigDB (version 1.0, https://dsigdb.tanlab.org) gene-drug association database using Se-ASRGs as the input gene set. Enrichment results were filtered based on adjusted *P*-values and visualized as dot plots, where dot size represents the number of overlapping genes and color intensity reflects enrichment significance. A gene-drug interaction network was subsequently constructed, with Se-ASRGs as core nodes, database-supported drugs or compounds as interaction nodes, and edges representing gene-drug associations derived from existing databases or literature.

### 2.9 Animal experiments

#### 2.9.1 Mice

Male C57BL/6 mice were purchased from Vital River (Beijing, China) and allocated to two age groups: young (2 months) and aged (19–22 months). Animals were maintained in pathogen-free housing under a 12-h light/dark cycle at 22 ± 2 °C and 50–60% relative humidity, with ad libitum access to food and water. Experimental procedures were approved by the Animal Welfare and Ethical Committee of Tianjin Medical University General Hospital (Approval Number: IRB2023-DWFL-051) and conducted in accordance with institutional ethical guidelines.

#### 2.9.2 Western blot analysis

Animals were euthanized with isoflurane, and brains were rapidly dissected on ice; the cerebral cortex was collected and snap-frozen in liquid nitrogen. Cortical tissue was homogenized in RIPA buffer (50 mM Tris-HCl, pH 7.4, 150 mM NaCl, 1% NP-40, 0.5% sodium deoxycholate, 0.1% SDS) supplemented with protease and phosphatase inhibitors. Homogenates were centrifuged at 12,000 × g for 15 min at 4 °C, and supernatants were collected. Protein concentrations were determined using a BCA protein assay kit (PC0020, Solarbio, China) according to the manufacturer's instructions, and samples were adjusted to the desired concentration. Loading buffer was added at a 4:1 (sample: loading buffer) volume ratio, followed by gentle pipetting or brief vortexing. Samples were denatured at 100 °C for 10 min, resolved on 10% SDS-PAGE, and transferred to PVDF membranes. Membranes were blocked with 5% non-fat milk in TBST (TBS + 0.1% Tween-20) for 2 h at room temperature, then incubated overnight at 4 °C with primary antibodies: SP1 (rabbit, 1:800, Abclonal, China), SEPHS2 (rabbit, 1:1000, Proteintech, China), and β-actin (rabbit, 1:1000, Proteintech, China). After washing, membranes were incubated with HRP-conjugated secondary antibodies for 1 h at room temperature. Protein bands were visualized by enhanced chemiluminescence and imaged. Subsequently, densitometric analysis was performed on uncropped images by investigators blinded to group assignment. Images were analyzed using ImageJ software as previously described (Degasperi et al., 2014). Bands were converted to 8-bit grayscale, and the background was subtracted using the rolling-ball method. The integrated density of each band was measured, normalized to the corresponding β-actin value, and expressed as a fold change relative to the control group. Data are presented as mean ± SD (*n* = 5 per group).

#### 2.9.3 Quantitative real-time PCR (qRT-PCR)

Animals were euthanized with isoflurane, and brains were rapidly dissected on ice; the cerebral cortex was collected and snap-frozen in liquid nitrogen. Total RNA was extracted using a kit (ET121, TransGen Biotech, China) according to the manufacturer's protocol. cDNA was synthesized from 1 μg total RNA using the synthesis kit (AE311-02, TransGen Biotech, China). qPCR was performed using SYBR Green master mix (AS221, TransGen Biotech, China) on a real-time PCR system according to the manufacturer's protocol. Primer sequences are listed in Supplementary Table S1. Relative mRNA expression of selected selenium-related and aging-associated genes was normalized to β-actin (ACTB) and calculated using the 2^−ΔΔ^Ct method (Livak and Schmittgen, 2001). Data are presented as mean ± SD (*n* = 3 per group).

## 3 Results

### 3.1 General analysis

**Figure 1:**
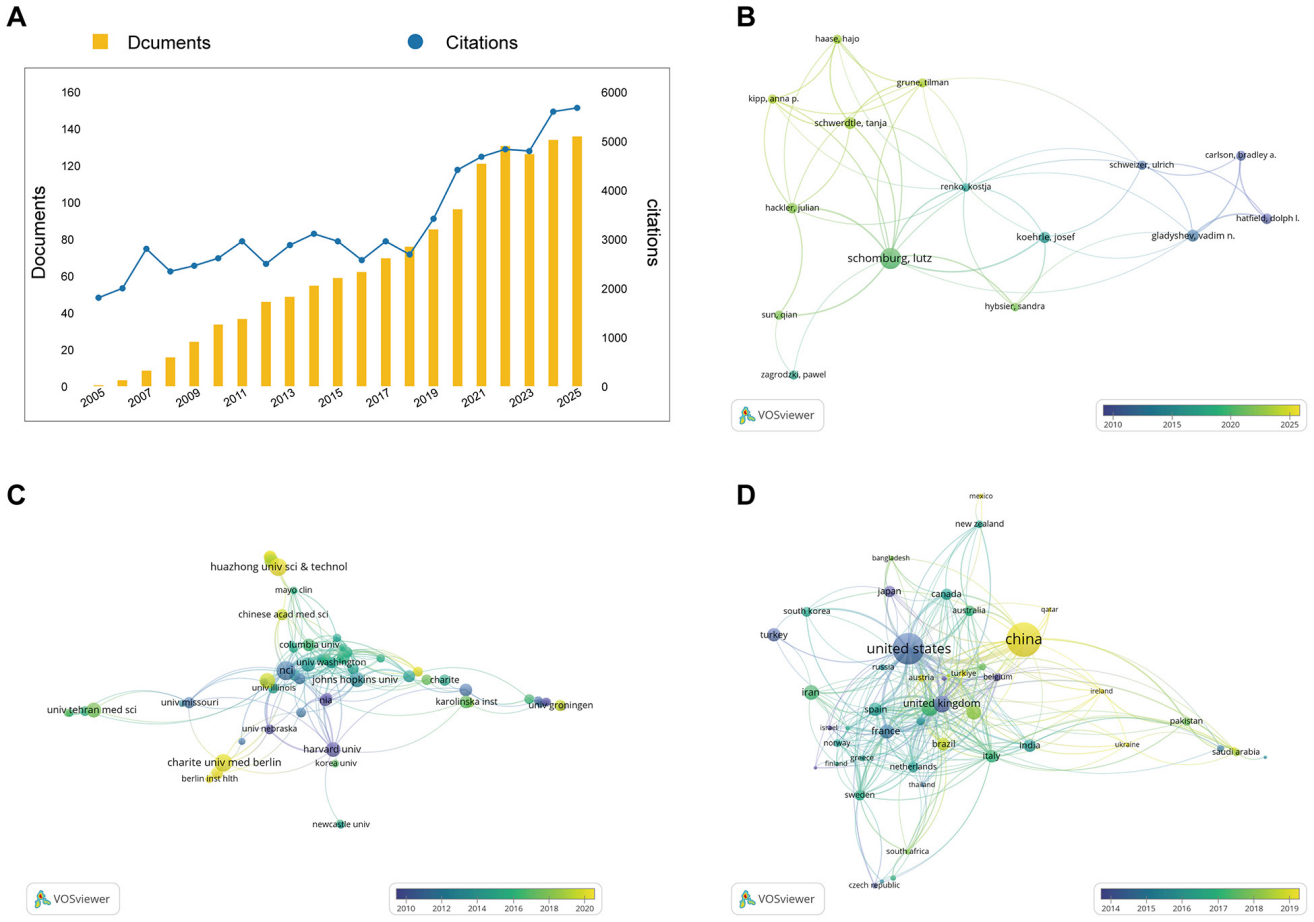
Bibliometric overview of selenium and aging research. **(A)** Annual publication output and citation trends from 2005 to 2025 based on original research articles indexed in the WoSCC. **(B)** The top 10 most productive authors ranked by number of articles. **(C)** The top 10 most contributing institutions ranked by publication output. **(D)** The top 10 most productive countries are ranked by the number of articles.

Over the past 20 years, 1,826 publications related to selenium and aging were retrieved from the WoS database. Annual publication output and citation trends were quantified using the WoS citation analysis tools (Figure 1A). From 2005 to 2025, annual publication counts increased steadily, accompanied by a continuous accumulation of citations. In total, these publications received 47,217 citations, with an average of 27.44 citations per article and an h-index of 90, indicating the development of a stable body of highly cited literature in this field.

Further analysis of academic output related to selenium and aging was conducted at the author, institutional, and national levels. Based on publication counts, the top 10 most productive authors, institutions, and countries were identified (Figures 1B–D and Supplementary Tables S2–S4). Over the past two decades, Lutz Schomburg has been the most prolific author in this field. Phyllis J. Goodman and Jack M. Guralnik, while having lower publication counts, were among the most highly cited contributors, especially in large-scale clinical studies and geriatric epidemiology. At the institutional level, institutions in the United States and Europe dominated both in publication output and citation frequency, whereas institutions from China exhibited high publication productivity. At the national level, research activity was concentrated in the United States and China, with substantial contributions from several European and emerging countries.

### 3.2 Journal landscape, intellectual base, and knowledge flow of selenium and aging research

**Figure 2:**
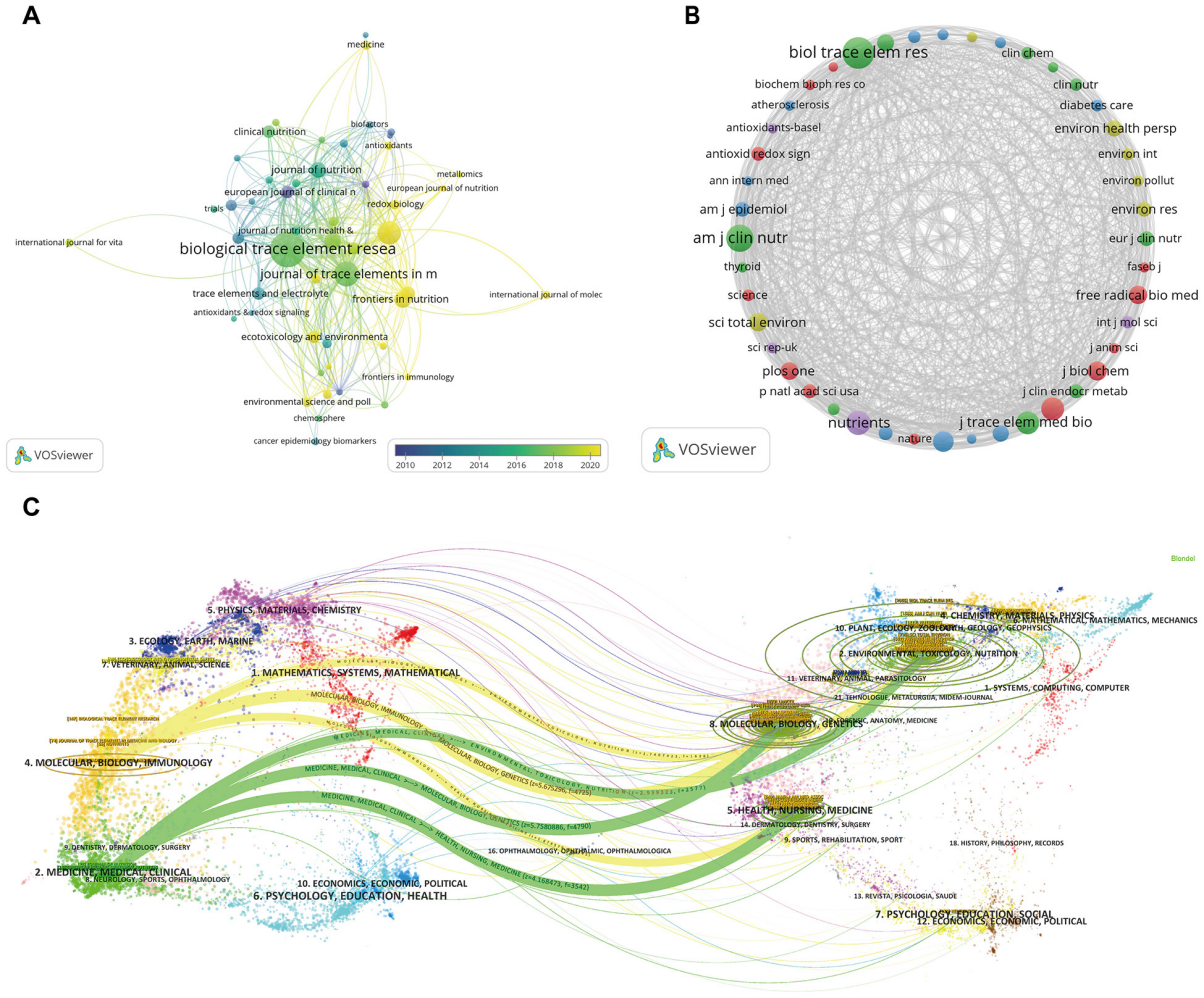
Journal-level citation analysis of selenium and aging research from 2005 to 2025. **(A)** Distribution of journals publishing selenium- and aging-related literature based on citation analysis. **(B)** The co-citation network of journals cited in this research field. **(C)** Bimap coverage of journals publishing literature on se and aging.

Journal-level citation analysis showed that research on selenium and aging was mainly published in journals focusing on trace elements and nutrition. *Biological Trace Element Research* and *Journal of Trace Elements* in Medicine and Biology had the highest publication output, representing the primary venues, while the top 10 journals are summarized in Supplementary Table S5. In contrast, Nutrition-related journals *(e.g., Nutrients, Journal of Nutrition, American Journal of Clinical Nutrition)* had moderate publication volumes but higher citation frequencies. Multidisciplinary journals such as *PLOS ONE* also facilitated cross-disciplinary dissemination (Figure 2A).

Co-citation analysis with a minimum threshold of 200 identified a core knowledge base in redox biology, molecular biology, environmental health, clinical nutrition, and epidemiology. Representative journals included *Free Radical Biology and Medicine, Antioxidants and Redox Signaling, Journal of Biological Chemistry, Environmental Health Perspectives, Science of the Total Environment, and American Journal of Clinical Nutrition* (Figure 2B).

Moreover, dual-map overlay revealed that studies were predominantly published in journals related to nutrition, clinical medicine, and environmental sciences, while cited journals clustered in molecular biology, redox biology, and environmental toxicology. Major citation pathways indicated a directional flow from basic and environmental research toward nutrition interventions, clinical applications, and studies on healthy aging (Figure 2C).

Together, selenium and aging research centers on trace element and nutrition journals, with a co-citation core linking basic biology to clinical and aging studies.

### 3.3 Evolving research landscapes of selenium and aging point to gaps in molecular mechanisms of brain aging

**Figure 3:**
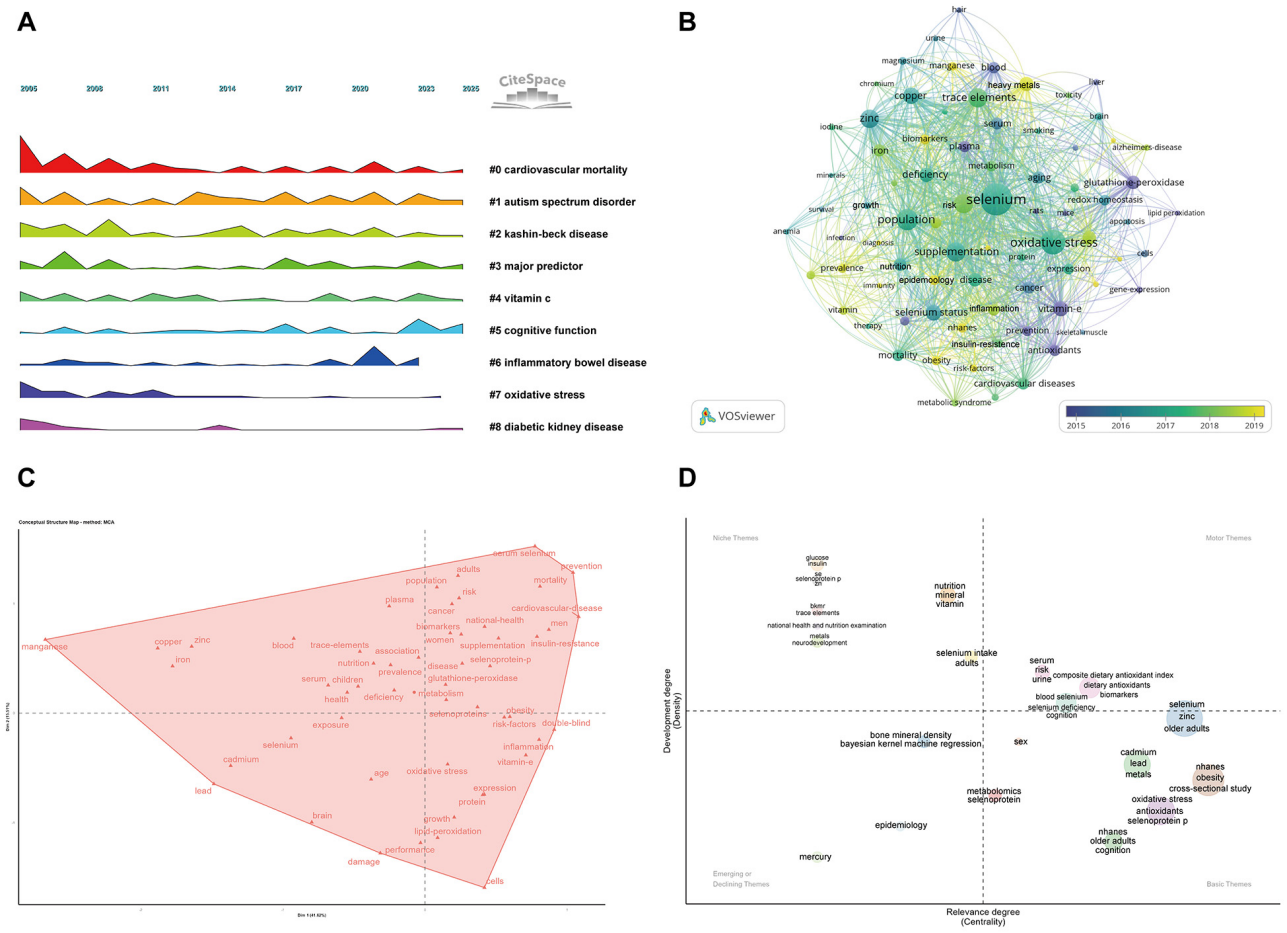
Bibliometric and thematic analysis of selenium and aging research. **(A)** Time-sliced keyword timeline showing the temporal distribution of selenium-related research themes. **(B)** Keyword co-occurrence network constructed using VOSviewer, with node size indicating keyword frequency. **(C)** Conceptual structure map based on multiple correspondence analysis in a two-dimensional semantic space. **(D)** Strategic thematic map showing the distribution of selenium and aging research themes by centrality and density.

Time-sliced keyword clustering analysis using CiteSpace revealed a clear temporal evolution of research themes related to selenium and aging (Figure 3A). During the early period (2005–2014), studies primarily focused on population-level health outcomes, particularly cardiovascular events and Keshan disease. As the field developed, the thematic scope broadened to include multiple chronic conditions, such as inflammatory disorders, metabolic diseases, and neurodevelopmental disorders. In recent years, topics related to cognitive function have remained consistently active and occupied stable positions within the keyword clusters.

Keywords co-occurrence analysis showed a high-density network centered on selenium and oxidative stress (Figure 3B), with high-frequency terms related to molecular mechanisms, including glutathione peroxidase, apoptosis, gene expression, and Alzheimer's disease. Temporal burst analysis of the top 30 keywords (Supplementary Figure 2) indicated a shift from early emphasis on antioxidant enzymes and lipid peroxidation to recent focus (2019–present) on selenoproteins, selenium metabolism, dietary antioxidants, and population-level risk factors, reflecting a move from mechanistic to epidemiological research.

Multiple correspondence analysis positioned selenium- and aging-related keywords evenly in a two-dimensional semantic space (Figure 3C). Selenium terms clustered near oxidative stress, lipid peroxidation, and gene expression, forming a molecular regulatory axis linked to brain function–related keywords. Thematic evolution analysis placed selenium, oxidative stress, selenoproteins, and cognition in the basic theme quadrant, with high centrality but limited internal development (Figure 3D). These patterns guided subsequent transcriptomic analyses of aging brain tissues.

### 3.4 Identification of aging-DEGs and functional enrichment analysis

**Figure 4:**
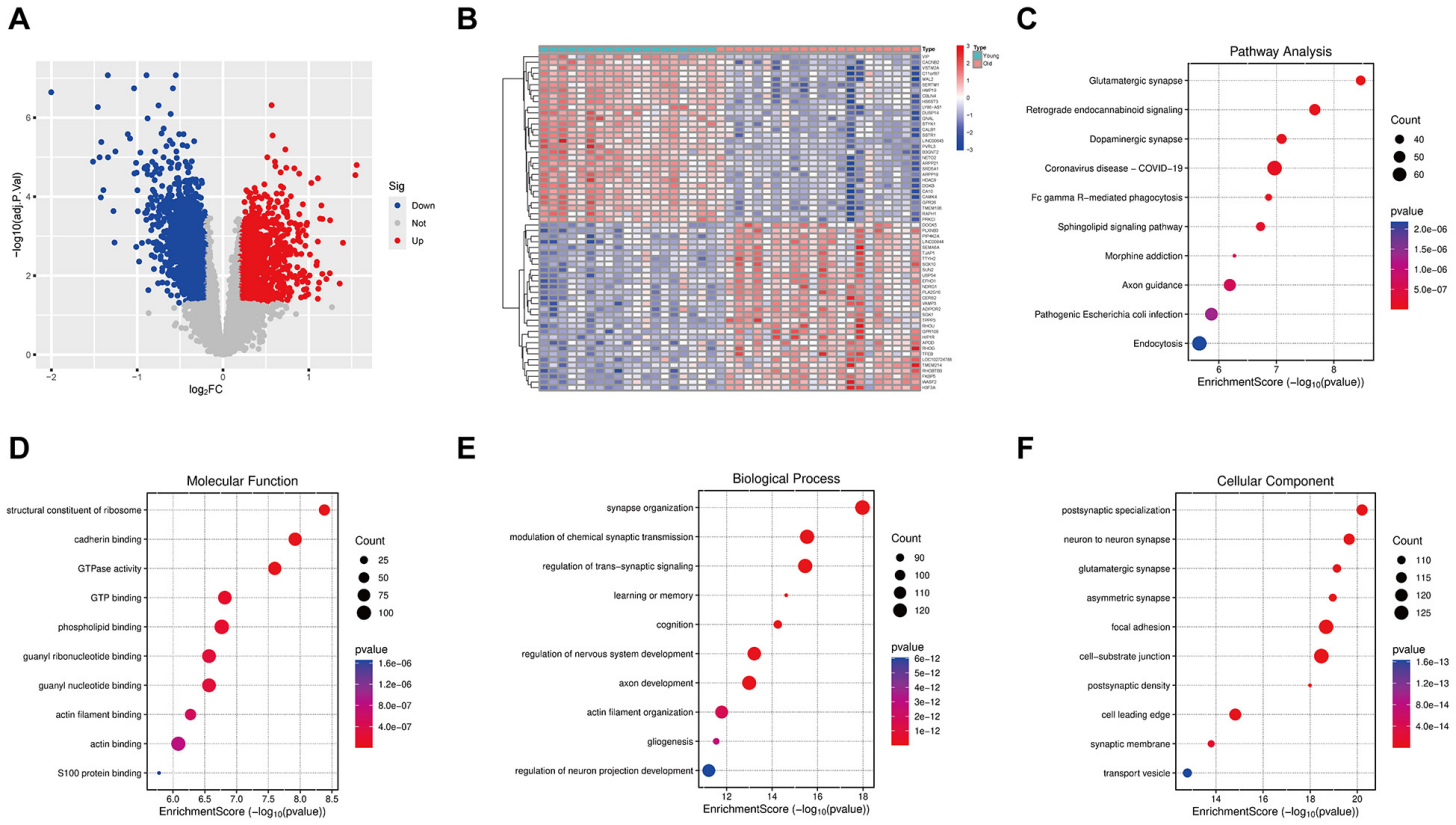
Differential expression and enrichment analysis in aging brain tissues. **(A)** Volcano plot of DEGs between the young and the aged brain tissues. **(B)** Heatmap of the top 30 upregulated and downregulated genes. **(C)** KEGG pathway enrichment of aging-related DEGs. **(D–F)** GO enrichment of DEGs across BP, CC, and MF categories.

To obtain aging-related differentially expressed genes (DEGs), the GSE53890 dataset was analyzed. A total of 3,064 DEGs were identified between aging and control brain tissues (Figure 4A). A heatmap (Figure 4B) showed the top 30 most significantly up- and downregulated genes between the old and young groups, with DEGs predominantly enriched in aging-related processes, including signal transduction and Ca^2+^ homeostasis, inflammation and immune regulation, epigenetic regulation, metabolic reprogramming, and cellular stress responses. KEGG pathway analysis revealed significant enrichment in synaptic and signaling pathways, such as glutamatergic synapse, dopaminergic synapse, and retrograde endocannabinoid signaling, as well as pathways related to axon guidance, endocytosis, and immune- and phagocytosis-associated processes (Figure 4C). Consistently, GO analysis demonstrated enrichment in biological processes including cellular stress response, immune regulation, and signal transduction, with cellular components mainly localized to extracellular and membrane-associated structures and molecular functions involving protein binding, receptor-ligand interactions, and enzymatic activities (Figures 4D–F).

### 3.5 Identification of hub Se-ASRG-related genes via machine learning

**Figure 5:**
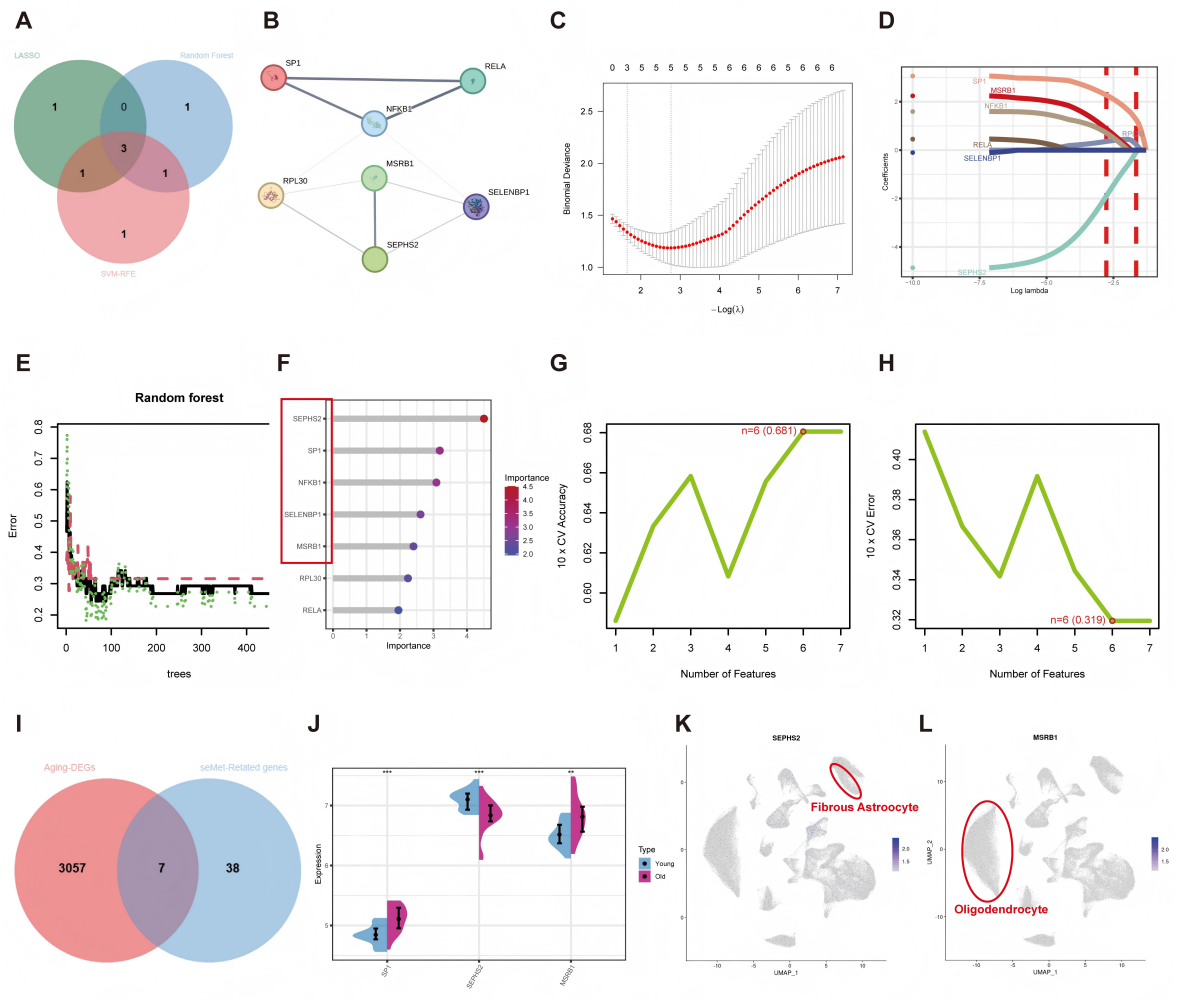
Identification of hub Se-ASRG-related genes via machine learning. **(A)** Overlap of aging-related DEGs and selenium-related genes identifying candidate Se-ASRGs. **(B)** PPI network of candidate Se-ASRGs. **(C, D)** LASSO coefficient analysis with optimal lambda (vertical dashed lines) determined via five-fold cross-validation. **(E, F)** Random forests: relationship between tree number and error rate, ranking of Se-ASRGs. **(G, H)** SVM-RFE algorithm's maximum accuracy and minimum error plots. **(I)** The Venn diagram of overlapping genes among LASSO, SVM-RFE, and Random Forest. **(J)** Differential expression of three hub Se-ASRGs between young and old groups. **(K, L)** Cell-type distribution of SEPHS2 and MSRB1 in the DISCO single-cell transcriptomic database.

The overlap between selenium-related gene sets and aging-DEGs (Se-ASRGs) was identified, from which seven Se-ASRGs were obtained (Figure 5A). To further explore the functional relationships among Se-ASRGs, a PPI network was constructed from the STRING database (Figure 5B). Three machine learning algorithms were then applied to screen hub Se-ASRGs. LASSO analysis identified five feature genes (Figures 5C, D). In the Random Forest analysis, five feature genes were selected using an importance score threshold greater than 2.5 (Figures 5E, F). SVM-RFE retained six feature genes (Figures 5G, H). Combined analysis of the three algorithms consistently identified three hub Se-ASRGs: SP1, SEPHS2, and MSRB1 (Figure 5I). To further characterize the biological features of these hub Se-ASRGs, chromosomal localization analysis showed that SEPHS2 and MSRB1 were located on chromosome 16, whereas SP1 was located on chromosome 12 (Supplementary Figure 3). Figure 5J showed differential expression of the three hub Se-ASRGs between the young and old groups. Moreover, cellular localization data from the DISCO (Li et al., 2022) database (https://www.immunesinglecell.org/) indicated that SEPHS2 expression was primarily observed in fibrous astrocytes, and MSRB1 was predominantly enriched in oligodendrocytes (Figures 5K, L).

### 3.6 SHAP analysis

**Figure 6:**
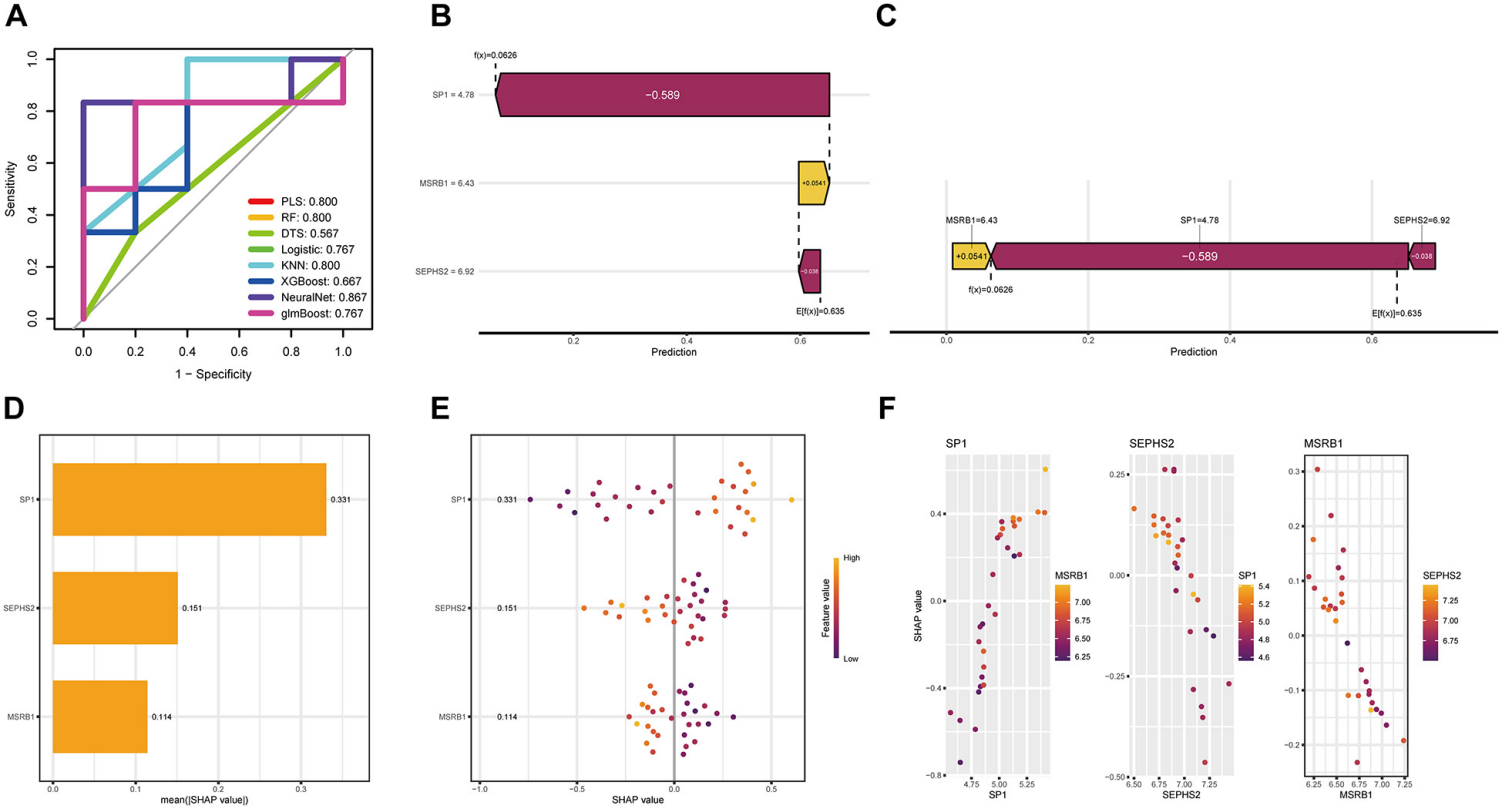
Interpretability analysis of hub Se-ASRGs. **(A)** Comparative performance of eight machine learning models for the hub gene. **(B, C)** SHAP waterfall and force plots illustrating aggregated effects of hub gene on single-sample predictions. **(D)** Feature importance bar plot ranking hub gene contributions. **(E)** SHAP beeswarm plot displaying SHAP value distributions. **(F)** Scatter plots demonstrating associations between gene expression and SHAP values.

To interpret the black-box nature of our machine learning-derived gene prioritization and identify key biological drivers, we employed SHapley Additive exPlanations (SHAP) analysis. First, we evaluated the performance of eight machine learning models using ROC curves (Figure 6A). The neural network model achieved the highest area under the curve (AUC = 0.867), followed by partial least squares (PLS), random forest (RF), and K-nearest neighbors (KNN), all with an AUC of 0.800. This identified the neural network as the most robust model for subsequent interpretative analysis.

SHAP analysis revealed the relative importance and directional effects of the top three features: SP1, SEPHS2, and MSRB1. The mean absolute SHAP values (Figure 6D) ranked SP1 as the most influential feature (mean |SHAP| = 0.331), followed by SEPHS2 (0.151) and MSRB1 (0.114). A force plot for a representative sample (Figures 6B, C) illustrated that SP1 exerted a strong negative contribution (SHAP = −0.589) to the final prediction, outweighing the minor positive effect of MSRB1 (SHAP = +0.0541) and negative effect of SEPHS2 (SHAP = −0.038), yielding a prediction [f(x) = 0.0626] that was markedly lower than the baseline expectation {E[f(x)] = 0.635}. The SHAP summary plot (Figure 6E) further elucidated feature-dependent effects. For SP1, high expression levels (depicted by yellow points) were predominantly associated with positive SHAP values, whereas low expression (purple points) correlated with negative SHAP values, indicating that SP1 upregulation tends to drive predictions in a positive direction. Similar directional trends were observed for SEPHS2 and MSRB1. Dependency plots (Figure 6F) uncovered significant synergistic interactions: high expression of SEPHS2 (depicted in yellow) consistently amplified the positive SHAP effects of both SP1 and MSRB1, suggesting a cooperative role within the predictive framework.

Based on their superior importance scores, consistent directional effects, and significant interactive contributions, SP1 and SEPHS2 were prioritized as hub genes for subsequent predictive model construction and clinical validation analyses.

### 3.7 Nomogram model performance

**Figure 7:**
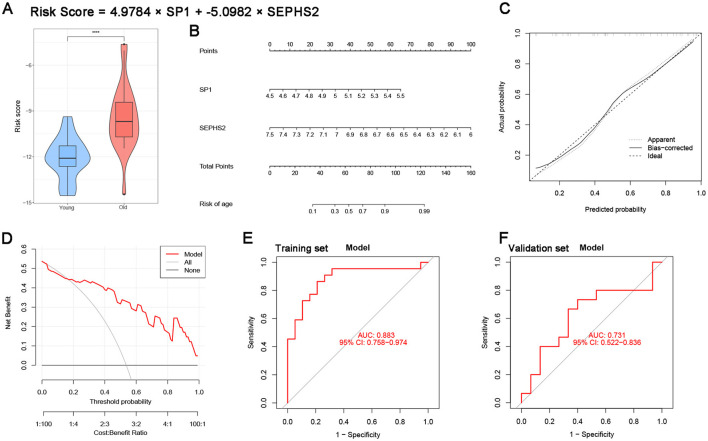
Nomogram model performance. **(A)** Violin plot of risk in the old and young groups. **(B)** Nomogram for predicting age-related risk based on SP1 and SEPHS2 expression. **(C)** Calibration curve of the nomogram. **(D)** DCA of clinical net benefit. **(E)** ROC curve in the training set. **(F)** ROC curve in the external validation set GSE1572.

We constructed a two-gene nomogram based on the hub genes SP1 and SEPHS2 to predict age-related risk, with the risk score calculated as: Risk Score = 4.9784 × SP1 −5.0982 × SEPHS2. The nomogram (Figure 7B) translates the expression levels of SP1 and SEPHS2 into a total point score, which directly maps to a predicted probability of age-related risk, providing a clinically actionable tool for individualized risk stratification. Violin plot analysis (Figure 7A) revealed that the risk score was significantly higher in the “Old” group compared to the “Young” group (p < 0.0001), confirming the model's ability to effectively distinguish between age groups.

Calibration curve analysis (Figure 7C) demonstrated excellent agreement between the predicted and actual probabilities, as the bias-corrected curve closely aligned with the ideal diagonal line, indicating high calibration accuracy of the nomogram. Decision curve analysis (DCA, Figure 7D) further supported the clinical utility of the model: across a wide range of threshold probabilities (0 to 0.7), the nomogram yielded a greater net benefit than either treating all patients (All) or none (None), highlighting its potential to improve clinical decision-making.

In the training set, the nomogram achieved an area under the receiver operating characteristic curve (AUC) of 0.883 (95% CI: 0.758–0.974; Figure 7E), reflecting excellent discriminatory power. To validate the generalizability of our findings, we tested the model on an independent external dataset, GSE1572. In this validation set, the nomogram maintained a robust AUC of 0.731 (95% CI: 0.522–0.836; Figure 7F), confirming its ability to generalize to new patient cohorts.

Collectively, these results demonstrate that the SP1/SEPHS2 nomogram represents a reliable and clinically useful tool for predicting age-related risk, with strong discriminatory performance, good calibration, and meaningful net clinical benefit, further validating the critical role of these two hub genes in our predictive framework.

### 3.8 Immune microenvironment characteristics associated with hub Se-ASRGs

**Figure 8:**
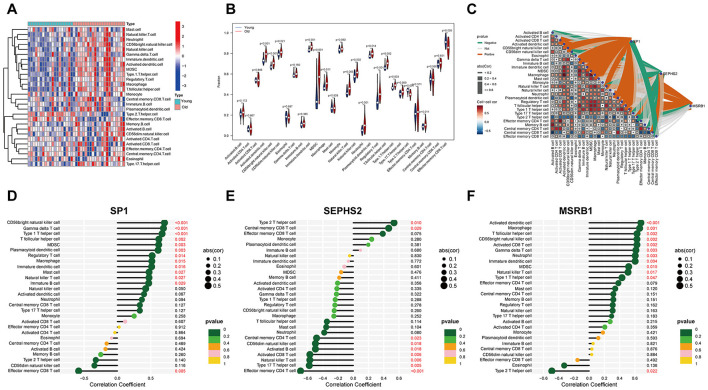
Immune cell infiltration analysis. **(A)** Heatmap showing the relative proportions of 22 immune cell types in the Young and the Old groups. **(B)** Violin plots showing differences in immune cell infiltration between the Young and the Old groups. **(C)** Correlation matrix and network visualization showing associations between hub gene expression and immune cell proportions. **(D–F)** Lollipop plots illustrating correlations between immune cell proportions and expression of SP1 **(D)**, SEPHS2 **(E)**, and MSRB1 **(F)**.

Comparisons of immune cell infiltration between the young and the old samples identified significant alterations in the relative abundances of multiple immune cell populations. Specifically, the old group showed significantly higher proportions of CD56^∧^bright NK cells (*p* < 0.001), CD56^∧^dim NK cells (*p* ≈ 0.001), monocytes (*p* < 0.001), macrophages (*p* = 0.001), neutrophils (*p* ≈ 0.014), and MDSCs (*p* = 0.002) compared with the Young group (Figures 8A, B). Correlation network analysis further revealed relationships between hub gene expression levels and immune cell proportions within the immune cell correlation network (Figure 8C). SP1 expression showed significant associations with CD56bright natural killer cells and effector memory CD8 T cells (Figure 8D and Supplementary Figure 4A). SEPHS2 expression was correlated with effector memory CD4 T cells and type 2 T helper cells (Figure 8E and Supplementary Figure 4B), while MSRB1 expression demonstrated significant correlations with activated dendritic cells and type 2 T helper cells (Figure 8F and Supplementary Figure 4C). Collectively, these findings provide key insights into the interactions between hub Se-ASRGs and specific immune cell subsets in the aging brain.

### 3.9 Translational validation of hub Se-ASRGs by drug prediction and experimental verification

**Figure 9:**
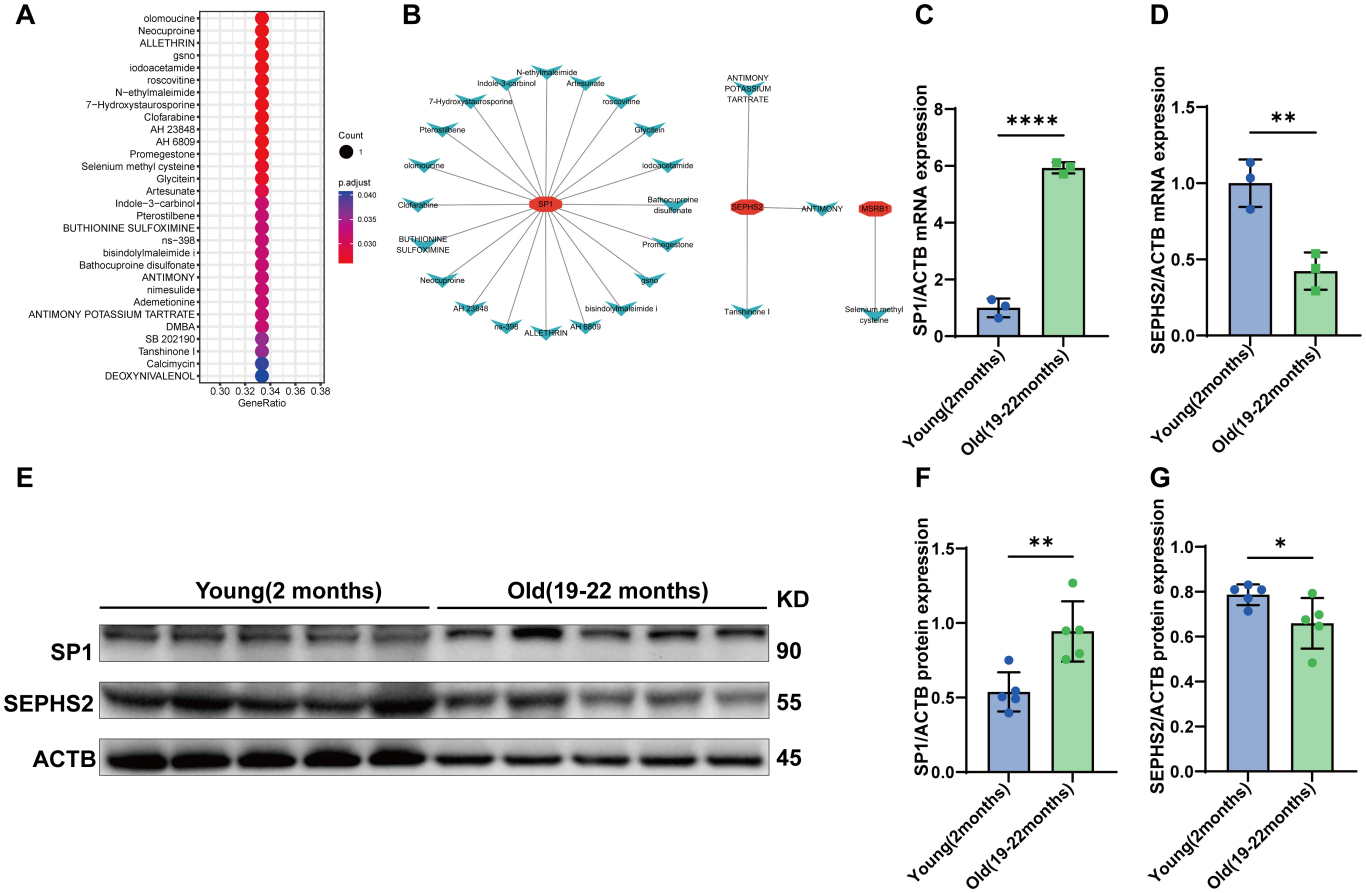
Prediction of drug associations and experimental validation. **(A)** Drug enrichment dot plot of hub Se-ASRGs. **(B)** Gene-drug interaction network of hub Se-ASRGs. **(C, D)** qPCR analyses (*n* = 3) and **(E–G)** Western blot analyses (*n* = 5) with corresponding quantification for validating SP1 and SEPHS2 expression in the animal model. Data are presented as mean ± SD. Statistical analyses were performed using two-tailed Student's t-tests. Statistical significance is indicated as follows: *p < 0.05, **p < 0.01, ***p < 0.001, ****p < 0.0001.

To analyze drug associations of hub Se-ASRGs, drug enrichment prediction and experimental analysis were performed. Dot plot analysis showed that multiple small-molecule compounds were significantly enriched in pathways associated with hub genes, including cell cycle regulation, oxidative stress responses, and thiol metabolism (Figure 9A). The gene-drug interaction network highlighted SP1's connections with multiple small molecules regulating the cell cycle and modulating oxidative stress pathways (Figure 9B). SEPHS2 displayed a more specific drug association pattern, primarily involving selenium donor-related compounds. PCR analysis of the cerebral cortex in young (2 months) and aged (19–22 months) mice revealed a significant increase in SP1 expression (*P* < 0.0001) and a significant decrease in SEPHS2 expression (*P* < 0.01) (Figures 9C, D). These transcriptional changes were further corroborated at the protein level by Western blot analysis, which revealed significant increases in SP1 (*P* < 0.01) and decreases in SEPHS2 protein expression (*P* < 0.05) (Figures 9E–G).

## 4 Discussion

Selenium supplementation has long been considered beneficial for delaying the aging process and preventing age-related diseases (McCann and Ames, 2011; Bjørklund et al., 2022). However, accumulating evidence indicates that its protective effects are inconsistent, and excessive doses may be detrimental, fueling an ongoing debate regarding its role in human aging and associated pathologies (Maraldi et al., 2011; Cai et al., 2019). Despite this controversy, research interest and academic impact in this field have steadily increased over the past two decades (Figure 1A), highlighting its importance. It is well known that Se enhances the function of antioxidant enzymes and selenoproteins, thereby alleviating oxidative stress-mediated cellular damage and delaying cellular and systemic aging (Bjørklund et al., 2022; Daneshpour et al., 2025). Bibliometric analysis reveals that research themes concerning selenium and aging have gradually expanded from traditional redox biology into nutrition and clinical research. However, molecular network structures centered on core themes such as “oxidative stress,” “selenoproteins,” and “cognitive function” remain incompletely explored (Figures 2, 3), with a notable scarcity of molecular-level analyses utilizing human brain transcriptomic data. To address this gap, we integrated selenium-related genes with transcriptome data from the aging human brain and employed machine learning to identify SP1, SEPHS2, and MSRB1 as key selenium-associated brain aging signatures (Se-ASRGs). In contrast to conventional notions that primarily emphasize the antioxidant properties of selenium, this study maps the evolutionary trajectory of the selenium-aging field while elucidating key selenium-aging regulatory nodes from human brain transcriptomic data. Collectively, our findings not only establish a bibliometric foundation for identifying research priorities but also highlight explicit molecular targets for subsequent mechanistic validation and the development of targeted intervention strategies.

Specificity protein 1 (SP1) is a zinc finger transcription factor that mediates transcriptional regulation by binding to conserved promoter elements (Raiber et al., 2012) and functions as an important regulator of redox balance and stress responses in the nervous system (Kim et al., 2022). SP1 dysfunction has been recognized as a molecular marker associated with brain aging (Kim et al., 2012). Integrated analyses of transcriptomic and methylation data from the aging human brain indicate that SP1 and its downstream targets occupy central positions within aging-related regulatory networks (Meng et al., 2016). This network centrality is consistent with the direct transcriptional regulation of multiple selenoprotein genes by SP1 (Amantana et al., 2004; Sun et al., 2023), providing a molecular basis for its involvement in pathogenic pathways associated with Alzheimer's disease through regulation of antioxidant and metabolic processes (Meng et al., 2016; Zhang and Kiryu, 2023). Furthermore, developmental exposure to environmental toxicants has been shown to induce persistent SP1 upregulation during aging, accompanied by dysregulation of Alzheimer's disease-related genes, cognitive impairment, and tau pathology (Bihaqi and Zawia, 2013; Bihaqi et al., 2014). Notably, targeted modulation of SP1 has demonstrated neuroprotective effects in experimental models (Dong and Gao, 2024; Zhou et al., 2021). Our findings identify SP1 as a central regulatory node linking selenium-related molecular pathways with aging-associated transcriptional remodeling in the human brain. SP1 therefore appears to coordinate selenium homeostasis, oxidative stress responses, aging processes, cognitive decline, and Alzheimer's disease-associated transcriptional changes. Collectively, these results highlight the SP1-selenoprotein regulatory axis as a promising target for precision interventions aimed at preserving neurobiological function and mitigating age-related neurodegenerative processes.

Selenophosphate synthetase 2 (SEPHS2) is a key enzyme in selenocysteine biosynthesis that regulates selenium availability for selenoprotein synthesis (Xu et al., 2007). Recent studies have demonstrated that SEPHS2-mediated selenoprotein biosynthesis is critical for cellular stress adaptation (Carlisle et al., 2020), raising the possibility that SEPHS2 dysregulation may contribute to aging-related processes. Notably, a genome-wide association study involving 10,763 individuals reported an association between SEPHS2 and subjective cognitive decline, and Mendelian randomization analysis further demonstrated that SEPHS2 expression in blood and cerebellar tissues is associated with neurodegenerative disease progression (Wang et al., 2025). Our study further indicates that SEPHS2 functions as a key molecular link between selenium metabolism and brain aging. Consequently, SEPHS2 may represent a promising selenium-related target for mitigating aging processes and alleviating age-associated neurological disorders.

Although Methionine sulfoxide reductase B1 (MSRB1) was identified as a Se-ASRG, SHAP analyses and internal validation both demonstrated that other variables contributed far less to model discrimination than SP1 and SEPHS2; therefore, it was not prioritized for experimental validation. Nevertheless, MSRB1 is essential for maintaining cellular redox homeostasis by repairing oxidized proteins, and its expression is highly responsive to dietary selenium and declines with age in mice (Lourenço Dos Santos et al., 2018; Chandran and Binninger, 2023; Novoselov et al., 2010), suggesting that selenium imbalance or age-related downregulation may impair MSRB1 function and thereby accelerate oxidative stress accumulation. In addition, MSRB1 dysfunction can disrupt actin polymerization-dependent innate immune responses, potentially contributing to age-related immune decline under selenium deficiency (Lee et al., 2013; Avery and Hoffmann, 2018). Taken together, these findings suggest that, despite its relatively modest contribution to the predictive model, MSRB1 may still act as a functionally important mediator linking selenium status to the regulation of oxidative stress and immune homeostasis during aging.

An aging-risk nomogram was constructed based on SP1 and SEPHS2. The model exhibited robust discriminative performance in the study cohort (AUC = 0.885), underscoring its potential for the preliminary individualized assessment of biological aging risk. Immune infiltration analysis revealed that innate immune cells were predominantly enriched during the aging process, accompanied by alterations in specific adaptive immune subsets. Significant correlations were observed between the expression of SP1, SEPHS2, and MSRB1 and distinct immune cell populations. These findings suggest that selenium-related pathways may modulate aging–associated neuroimmune remodeling, potentially by influencing redox homeostasis and selenoprotein-mediated signaling transduction. Drug-enrichment analyses identified several candidate small molecules whose targets overlap with pathways involved in selenium metabolism and redox regulation, highlighting promising opportunities for targeted intervention and drug repurposing. Furthermore, experimental validation in animal models demonstrated that the expression levels of SP1 and SEPHS2 were consistent with the bioinformatic predictions.

However, this study has several limitations. Firstly, the bibliometric analysis was restricted to original English research articles retrieved from the WoSCC. While WoSCC ensures robust metadata consistency and bibliometric compatibility, this selection may introduce inherent source and language biases. Future research should integrate multilingual literature and diverse databases to enhance the comprehensiveness and representativeness of the findings. Secondly, as a retrospective analysis based on public datasets, this study is characterized by a relatively homogeneous study cohort. Prospective studies involving larger sample sizes and greater statistical power are essential to validate the clinical applicability and effectiveness of these results. Thirdly, the observational design of this study, together with the absence of longitudinal molecular measurements, prevents definitive causal inference regarding selenium, brain aging, and the molecular candidates identified. Subsequent investigations should employ Mendelian randomization or prospective cohorts to elucidate causal directions. Furthermore, functional validation through genetic manipulation and selenium intervention targeting candidate genes in cells and animal models is required to confirm the underlying mechanisms.

## 5 Conclusion

Our study maps the global research landscape of selenium and aging, identifying the countries, institutions, authors, and journals that have made major contributions over the study period. Bibliometric analysis further reveals that recent research has increasingly focused on selenium and selenoproteins, aging, and cognition. Importantly, we highlight SP1 and SEPHS2 as key selenium-related nodes in aging brain tissues. Collectively, these findings not only synthesize the current research status on selenium and aging but also provide a molecular perspective on brain aging, pointing to candidate targets and strategies for assessing selenium-related molecular dysregulation and potential interventions in age-related neurological disorders.

## Data Availability

The datasets presented in this study can be found in online repositories. The repository name and accession numbers are as follows: Gene Expression Omnibus (GEO), https://www.ncbi.nlm.nih.gov/geo/, GSE53890; GSE1572.
