# Supplementary material for: Integrative bibliometric and transcriptomic analyses identify selenium-associated molecular signatures in the aging brain
Source: Front Aging Neurosci. 2026 May 4;18:1791352. doi: 10.3389/fnagi.2026.1791352 (PMC13180909; doi:10.3389/fnagi.2026.1791352)
Supplement: Supplementary file 1 [file Data_Sheet_1.doc]

**Integrative bibliometric and transcriptomic analyses identify selenium–associated molecular signatures in the aging brain**

**Supplementary Materials:**

**Table S1 TI search queries and refinement procedure**

| Set | Results | Refinement |
| --- | --- | --- |
| 4 | 1826 | Refined by publication years: 2005–2025 |
| 3 | 2160 | Refined by languages: English |
| 2 | 2200 | Refined by document types: Article |
| 1 | 2362 | Topic: TI=(selenium OR selenoprotein* OR ("selenoprotein P" OR SELENOP) OR selenoenzyme* OR selenocysteine OR selenomethionine OR "dietary selenium" OR "selenium supplementation" OR "selenium deficiency") AND TI=(aging OR ageing OR senescence OR "cellular senescence" OR longevity OR lifespan OR healthspan OR gerontology OR geriatrics OR "age-related" OR "age associated")) OR (AB=(selenium OR selenoprotein* OR ("selenoprotein P" OR SELENOP) OR selenoenzyme* OR selenocysteine OR selenomethionine OR "dietary selenium" OR "selenium supplementation" OR "selenium deficiency") AND AB=(aging OR ageing OR senescence OR "cellular senescence" OR longevity OR lifespan OR healthspan OR gerontology OR geriatrics OR "age-related" OR "age associated")) OR (AK=(selenium OR selenoprotein* OR ("selenoprotein P" OR SELENOP) OR selenoenzyme* OR selenocysteine OR selenomethionine OR "dietary selenium" OR "selenium supplementation" OR "selenium deficiency") AND AK=(aging OR ageing OR senescence OR "cellular senescence" OR longevity OR lifespan OR healthspan OR gerontology OR geriatrics OR "age-related" OR "age associated"))) NOT TS=(soil OR plant OR crop OR algae OR geochem* OR wastewater OR fertilizer* OR feed* OR livestock OR poultry OR pig* OR cattle OR fish* OR food* OR biofortification OR fermentation OR yeast OR environment* OR contamination OR sediment* OR mining OR nanoparticle* OR nanomaterial* OR semiconductor* OR implant* OR biomaterial* OR scaffold* OR coating* OR corrosion OR degradation OR insect* OR arthropod* OR ecological OR "population dynamics" OR "analytical method*" OR "detection method*") NOT DT=(Meeting Abstract OR Letter OR "Conference Proceeding" OR "Editorial" OR "Book Chapter" OR "Note" OR "Short Survey")  Indexes = SCI-EXPANDED |

**Table S2 Top 10 most productive authors** **in Se-related Aging** Research

| Author | Articles | Citations | Author Contributions |
| --- | --- | --- | --- |
| Lutz Schomburg | 31 | 940 | The most prolific authors and key figures in selenium biology |
| Hassan Imran Afridi | 13 | 249 | Authors active in recent years |
| Richard D. Semba | 12 | 934 | Highly cited, epidemiology- and nutrition-oriented aging research |
| Phyllis J. Goodman | 11 | 1761 | Most highly cited; large clinical and cohort studies |
| Tasneem Gul Kazi | 11 | 246 | Elemental analysis and analytical methods |
| Vadim N. Gladyshev | 11 | 215 | Mechanism-oriented leading researcher in selenoproteins and aging |
| Tanja Schwerdtle | 11 | 758 | Toxicology and safety of trace elements |
| Jack M. Guralnik | 9 | 1690 | Epidemiology of aging and functional decline in older populations |
| Jack M. Guralnik | 9 | 695 | High-impact contributor in clinical trials and biostatistics |
| Urban Alehagen | 9 | 377 | Selenium and cardiovascular outcomes in aging |

**Table S3 Top10 contributing institutions in Se-related Aging Research**

| Rank | Organization | Articles | Citations | Country |
| --- | --- | --- | --- | --- |
| 1 | National Cancer Institute | 31 | 3675 | United States |
| 2 | Charité-Universitätsmedizin Berlin | 27 | 912 | Germany |
| 3 | Huazhong University of Science and Technology | 27 | 697 | China |
| 4 | Chinese Center for Disease Control and Prevention | 21 | 577 | China |
| 5 | Tehran University of Medical Sciences | 20 | 341 | Iran |
| 6 | Johns Hopkins University | 19 | 1417 | United States |
| 7 | Harvard University | 19 | 855 | United States |
| 8 | University of São Paulo | 19 | 298 | Brazil |
| 9 | Pomeranian Medical University | 18 | 380 | Poland |
| 10 | University of Michigan | 17 | 3654 | United States |

**Table S4 Top10 contributing countries in Se-related Aging Research**

| Rank | Articles | Articles | Citations |
| --- | --- | --- | --- |
| 1 | China | 461 | 8846 |
| 2 | United States | 383 | 18043 |
| 3 | United Kingdom | 117 | 5501 |
| 4 | Germany | 115 | 3704 |
| 5 | Iran | 88 | 1920 |
| 6 | Poland | 87 | 1306 |
| 7 | France | 77 | 2929 |
| 8 | Spain | 71 | 2579 |
| 9 | Turkey | 68 | 1725 |
| 10 | Brazil | 67 | 1107 |

**Table S5 Journal distribution of publications in Se-related Aging Research**

| Journal | articles | Citations | IF |
| --- | --- | --- | --- |
| Biological Trace Element Research | 147 | 3297 | 3.6 |
| Journal of Trace Elements in Medicine and Biology | 74 | 1801 | 3.5 |
| Nutrients | 65 | 1246 | 5 |
| Frontiers in Nutrition | 35 | 260 | 5.1 |
| PLOS ONE | 31 | 1060 | 2.6 |
| Journal of Nutrition | 28 | 621 | 3.8 |
| Scientific Reports | 23 | 260 | 3.9 |
| Trace Elements and Electrolytes | 19 | 72 | 0.2 |
| Ecotoxicology and Environmental Safety | 19 | 275 | 6.1 |
| European Journal of Clinical Nutrition | 18 | 564 | 3.6 |


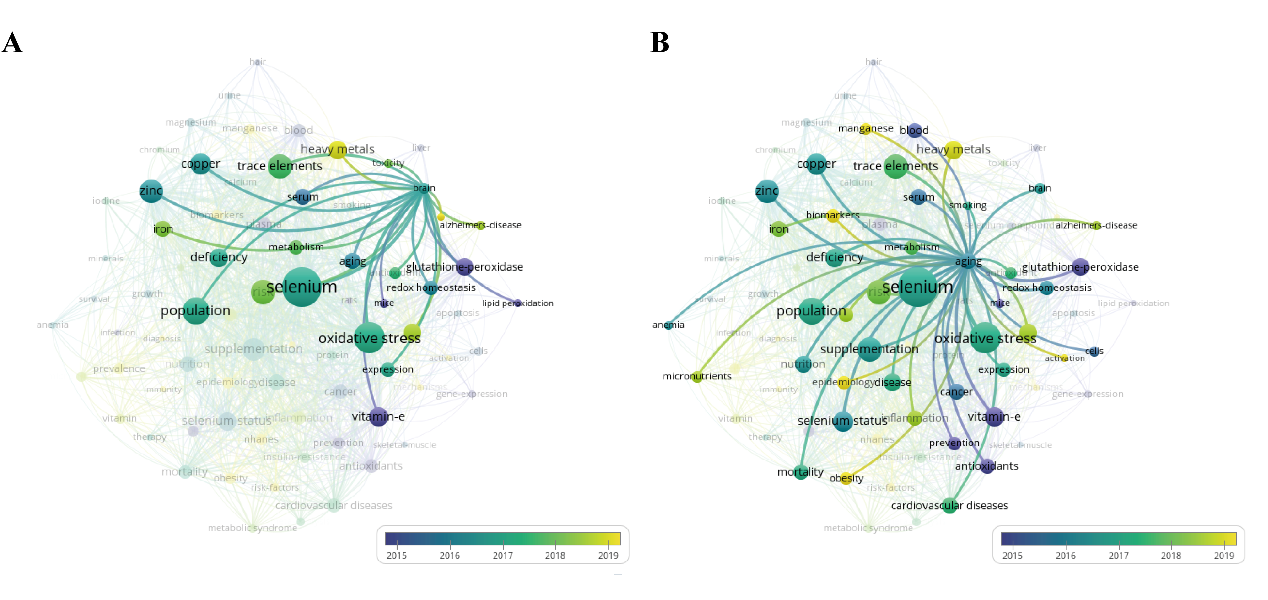


**Supplementary Figure 1A-B.** Brain- and aging-centered keyword co-occurrence networks in selenium-related research.


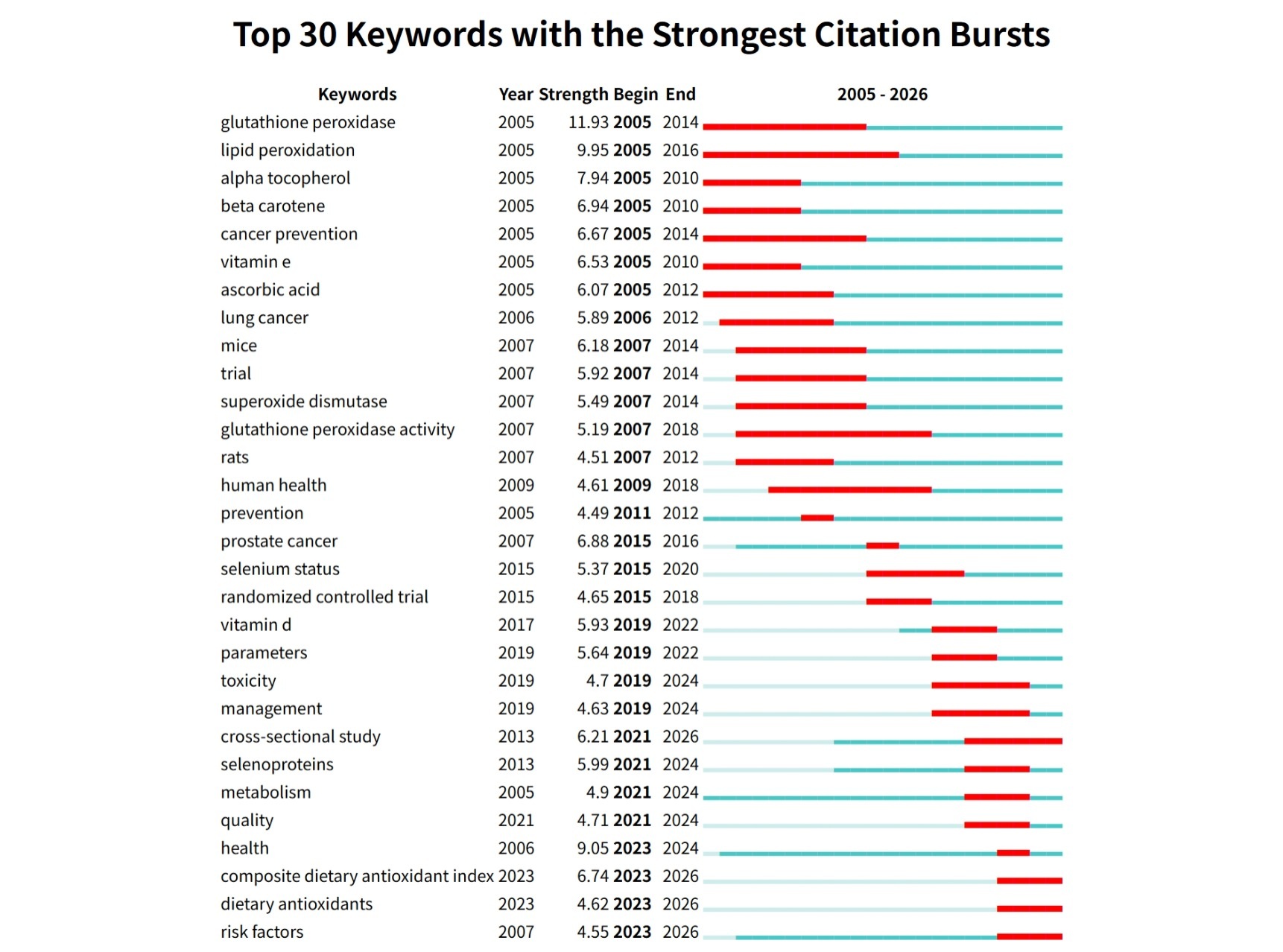


**Supplementary Figure 2.** Top 30 keywords for burst intensity and duration.


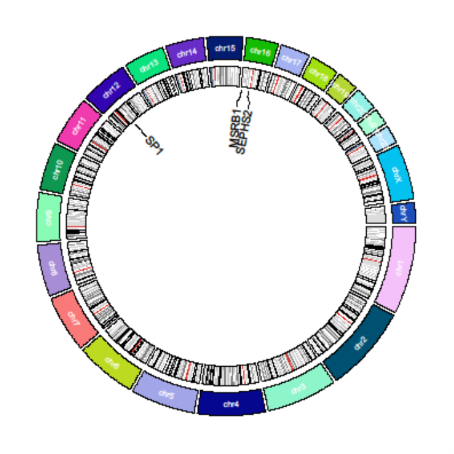


**Supplementary Figure 3.** Chromosome localization of hub Se-ASRG-related genes.


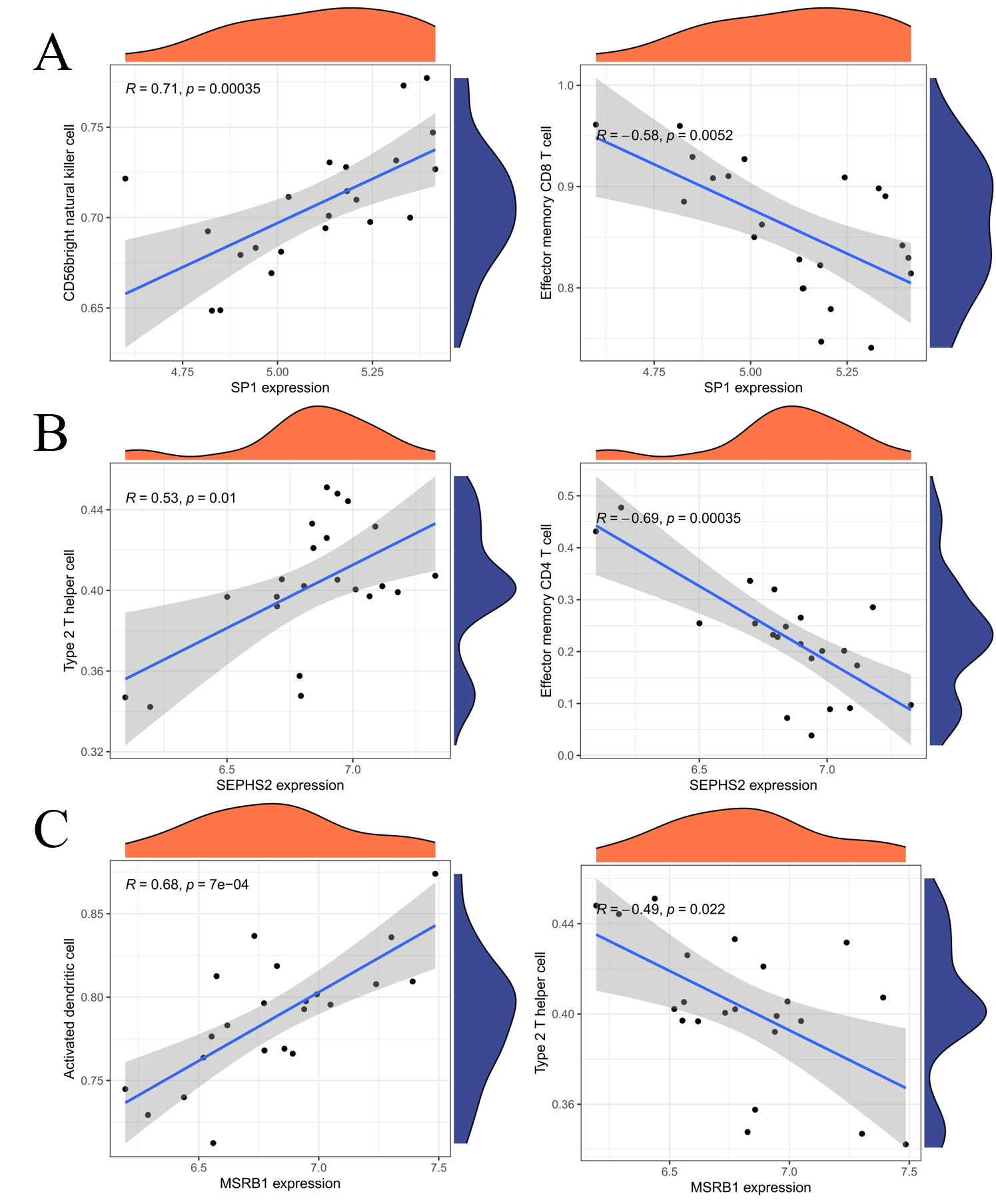


**Supplementary Figure 4.** (A–C) Scatter plots showing representative positively and negatively correlated immune cell types for SP1 (A), SEPHS2 (B), and MSRB1 (C).

**Abbreviations:** AUC, area under the curve; BP, biological process; CC, cellular component; CI, confidence interval; CIBERSORT, Cell-type Identification By Estimating Relative Subsets Of RNA Transcripts; DEGs, differentially expressed genes; DISCO, Database of Integrated Single-Cell Omics; DO, Disease Ontology; DSigDB, Drug Signature Database; DTS, decision tree structure; EPIC, European Prospective Investigation into Cancer and Nutrition; GEO, Gene Expression Omnibus; GO, Gene Ontology; KEGG, Kyoto Encyclopedia of Genes and Genomes; KNN, k-nearest neighbor; LASSO, least absolute shrinkage and selection operator; LLR, log-likelihood ratio; LR, logistic regression; MCA, multiple correspondence analysis; MDSCs, myeloid-derived suppressor cells; MF, molecular function; MSigDB, Molecular Signatures Database; MSRB1, methionine sulfoxide reductase B1; NK cells, natural killer cells; PLS, partial least squares; PPI, protein–protein interaction; qRT-PCR, quantitative real-time polymerase chain reaction; RF, random forest; ROC, receiver operating characteristic; Se, selenium; Se-ASRGs, selenium-associated aging-related genes; SEPHS2, selenophosphate synthetase 2;SP1,Specificity protein 1; SHAP, Shapley Additive exPlanations; SVM-RFE, support vector machine–recursive feature elimination; WB, Western blot; ACTB, β-actin. DCA, decision curve analysis; WoSCC, Web of Science Core Collection.
